# Systematic and quantitative analyses of hollow fiber model of *Mycobacterium abscessus* lung disease studies and new dosing recommendations

**DOI:** 10.1128/spectrum.03296-25

**Published:** 2026-04-21

**Authors:** Shashikant Srivastava, Tawanda Gumbo

**Affiliations:** 1 Division of Infectious Diseases, Department of Medicine, The University of Texas at Tyler School of Medicine 675071, Tyler, Texas, USA; 2 Department of Cellular and Molecular Biology, University of Texas Health Science Center at Tyler, Center for Biomedical Research 12341, Tyler, Texas, USA; 3 NASOS Biotechnologies, Dallas, Texas, USA; 4 IMPI Group of Companies, Mt. Hampden, Zimbabwe; 5 Phase Advance, Dallas, Texas, USA; ICON plc, London, United Kingdom

**Keywords:** new approach methodologies, inhalational formulations, sulbactam-durlobactam-ceftriaxone, epetraborole, omadacycline

## Abstract

Guideline-based combination therapy achieves sputum culture conversion rates in 23%–34% of patients with *Mycobacterium abscessus*-complex lung disease. Thus, new therapies are needed. We performed a systematic review to validate and benchmark the hollow fiber system model of *M. abscessus* lung disease for drug development. We performed a literature search to identify all published hollow fiber system pharmacokinetic-pharmacodynamic studies. Preferred Reporting Items for Systematic Reviews and Meta-Analyses was used for bias minimization. A total of 12 studies were identified. The average quality score was 13.7 out of 21. Eight were monotherapy (exposure-effect and dose-fractionation), one double β-lactam, and three guideline-based therapy studies. For omadacycline and imipenem, hollow fiber system data were accompanied by clinical real-world evidence confirmation. Microbial kill was always terminated by antimicrobial resistance. We used quantitative analyses to rank drugs’ efficacy based on CFU/mL kill below day 0 bacterial burden normalized to multi-drug guideline-based therapy kill. The highest-ranked drugs were sulbactam-durlobactam-ceftriaxone (177-fold), epetraborole (15-fold), and omadacycline (7-fold) better than guideline-based therapy. We used the target exposures identified in the systematic analysis in Monte Carlo experiments to identify optimal doses for inhaled formulations. The optimal inhalational dose of imipenem was 250 mg/day, for tigecycline 4 mg/day, for cefoxitin 50 mg/day, and for amikacin liposome inhalation suspension 590 mg once weekly. The hollow fiber system model of *M. abscessus* lung disease is tractable for exposure-effect, dose-fractionation, and factorial design combination studies. It could also be used to rank drugs and inform on which drugs to test in novel combinations.

**IMPORTANCE:**

Current treatments for *Mycobacterium abscessus* lung disease fail in 70%–80% of patients and are toxic. The hollow fiber system has been used to study old and new potential treatments for this disease. We performed a systematic review of this methodology, for lessons learned. We found 12 studies, which were of adequate quality. Efficacy was always terminated by antimicrobial resistance. The top three drugs in terms of efficacy were sulbactam-durlobactam-ceftriaxone, epetraborole, and omadacycline, which were 7 to 177 times better than standard of care. These drugs could be combined into a new treatment regimen better than current treatments. We also calculated new doses for imipenem, tigecycline, cefoxitin, and amikacin when administered as inhalational therapy. The inhaled doses were multiple-fold lower than intravenous ones, which could be less toxic. The hollow fiber system model is an easily managed system from drug development and dose finding for *M. abscessus* lung disease.

## INTRODUCTION

*Mycobacterium abscessus* (MAB) complex is responsible for 12% of all non-tuberculous mycobacteria (NTM) lung disease (LD) in the United States but could contribute up to 42% of NTMs elsewhere (1, 2). In one large study from Spain, MAB subspecies *abscessus* accounted for 52%, MAB subsp. *massiliense* for 34%, and MAB subsp. *bolletii* for 14% of all isolates (3). Guideline-based therapy includes an initial phase of treatment of at least three active drugs, based on the susceptibility testing, including one to two from the group of injectables such as amikacin or β-lactams (imipenem or cefoxitin) or tigecycline and two from orally formulated drugs such as clarithromycin (if no inducible resistance), or clofazimine or linezolid (4). A meta-analysis of 19 clinical studies demonstrated that guideline-based therapy achieved sputum culture conversion in only 34% of patients with MAB subsp. *abscessus* and 54% infected with other MAB *subspecies* as initial therapy; for salvage therapy, the sputum culture conversion was 23% for all subspecies (5). This means that it will be important to study these multiple subspecies when developing new therapies. In addition, the US Food and Drug Administration (FDA) and European Medicines Agencies (EMA) recently released roadmaps of New Approach Methodologies that “offer the tools to assess safety, efficacy, and pharmacology of drugs and therapeutics *without* traditional animal models” (6, 7). We (including Dr. Beatriz Ferro) designed the hollow fiber system model of MAB (HFS-MAB)-LD, which in tandem with Monte Carlo experiments (MCE), fits that designation of New Approach Methodologies, to test new therapies for MAB-LDs (8).

One of the most important factors associated with guideline-based therapy failure is the presence of cavities; those with diameter greater than 2 cm have odds ratios of 2.4 for failure to cure and 2.5 for death (9, 10). Thirty-five percent of patients had a median diameter of 3 cm (9). Lung cavities and nodules represent a physical barrier to antibiotics; concentrations fall across the gradient inversely proportional to the diameter (11, 12). Lung cavities also represent higher MAB burdens, with pre-treatment bacterial burdens (*B_0_*) in the range of 10^7^–10^8^ CFU/lung in patients (10, 13). Large bacterial burdens are associated with poorer outcomes in patients (10, 14–17). These clinicopathological features reflect the most important pharmacokinetic (PK) and pharmacodynamic (PD) factors that affect therapy outcomes such as inoculum effect and exposures achieved at site of infection (10–21). These PK/PD and clinicopathological features and human-like intralesional PKs were made part of the basic design of the HFS-MAB. Here, we performed a systematic review of this new approach methodology (NAM), for lessons learned, and to improve testing of new drugs for MAB-LD.

## MATERIALS AND METHODS

### Objectives and study questions

The first objective was to perform a systematic review and quantitative analysis to validate and benchmark the HFS-MAB for drug development, to be used in the future for voluntary exploratory data submissions to the FDA and EMA (6, 7, 19, 22–27). The second objective was to rank new/repurposed drugs based on the extent of microbial kill to design a new regimen. The third objective was to use the systematic review PK/PD findings to update dosing of new formulations and their susceptibility breakpoints. The fourth objective was to provide a first step for standardization of HFS-MAB model between laboratories.

### Minimal criteria for accepting studies

In antimicrobial PK/PD science, microbial kill modeling uses the four-parameter inhibitory sigmoid maximal effect (*E*_max_) model. The model has the following parameters: (i) *E*_con_ is the bacterial burden in nontreated controls, (ii) *E*_max_ is maximal effect or efficacy, (iii) *H* is the Hill slope, and (iv) EC_50_ is the drug exposure mediating 50% of *E*_max_ or potency (28). The equation is as follows:


(1) $${\text{Effect (log}}_{10}\text{ CFU/m)}=E_{\text{con}}-E_{\max}*EC^{H}/(EC^{H}+EC_{50}^{H})$$

Since this model has four parameters, at a minimum 4 + 1 doses or exposures are required for the model to have a unique solution, and this is the minimum definition for an acceptable exposure-response HFS-MAB study. The antibiotic resistance arrow of time has three parameters, so the five parameters required for equation 1 will be enough for identification of the drug exposure associated with antimicrobial resistance (AMR) suppression (29, 30).

### Quality scores

Recently, we developed a quality scoring tool for *Mycobacterium avium* complex preclinical PK/PD models, which we used here without modification (31). The score is categorized as a high score if >20, good score if 15–20, adequate if 10–14, poor quality if 5–9, and deficient and unreliable if <5.

### Literature search

Two authors (T.G. and S.S.) searched PubMed, Google Scholar, Interscience Conference on Antimicrobial Agents and Chemotherapy abstracts, ID Week abstracts, and Biorxiv for studies published through 1 August 2025. The last search was on 1 August 2025. We utilized the medical subject heading (MeSH) “hollow fiber” AND “*Mycobacterium abscessus*” for our search. We also included manuscripts we had submitted to journals and had been accepted for publication (our internal databases), even if not yet on PubMed. The two authors independently extracted the data into the prespecified table format, and then, each independently scored the studies for quality criteria, as described below. Consensus was reached for study inclusion after the discussion of each study. There was no exclusion of articles by language. Bias minimization was according to Preferred Reporting Items for Systematic Reviews and Meta-Analyses (PRISMA) (https://www.prisma-statement.org) (32).

### Data and outcomes collected

Data collected included number of HFS-MAB units, replicates used, number of doses tested, type of study design (exposure-effect or dose fractionation or factorial design), number of non-American Type Culture Collection (ATCC) clinical isolates tested, and number of doses in MCEs. The outcomes recorded were day zero microbial burden (*B*_0_) achieved, CFU/mL, microbial kill below *B*_0_, PK/PD parameter linked to efficacy and AMR suppression, the exposure mediating 80% of *E*_max_ (EC_80_), and MCEs based in *in silico* dose-ranging.

### Data synthesis and analyses

First, we performed a qualitative analysis of lessons learned, including PK/PD parameters linked to effect, emergence of AMR, and duration of the HFS-MAB experiments. This approach is descriptive. The reliability of each study was qualified by the quality score.

Second, we performed a quantitative analysis that calculated the extent to which a drug killed below *B*_0_ when at *E*_max_ in CFU/mL as a fold difference with the three-drug guideline-based therapy. This was calculated as follows:


 (2) $$\Delta B_{A}=B_{0}-B_{E_{\text{max}}}$$

(3) $$Fold\;difference=\Delta B/\Delta B_{GBT}$$

where 𝚫*B*_A_ is bacterial burden kill for test drug A below *B*_0_ and 𝚫*B*_GBT_ is microbial kill below *B*_0_ of combination guideline-based therapy in the HFS-MAB. Since one of the major drivers of quality score was the number of non-ATCC isolates used, microbial kill was inversely weighted by the number of non-ATCC isolates used in the study assuming 4 based on four to five isolates recommended by the EMA (31, 33). Thus, if four isolates were used, fold change was divided by 4/4; if only three were used, it was divided by 4/3. The ranking was then made from highest fold difference to the lowest. We also captured the EC_80_ PK/PD target for each study and PK/PD susceptibility breakpoints from published MCE, where available.

### MCEs for inhalational therapy

A recent approach to achieve the PK/PD exposure target for cefoxitin, tigecycline, amikacin, and imipenem/cilastatin (herein “imipenem”), hitherto administered intravenously, is inhalational therapy formulations (34–41). Here, we performed MCEs to identify inhalational therapy doses for drugs achieving or exceeding EC_80_ in epithelial lining fluid (ELF). Based on receptor theory and antibiotic as a ligand occupying its target (e.g., clarithromycin binding to subunit 50S of the bacterial ribosome), a saturable process, a drug cannot kill more than its *E*_max_ even if the exposure is increased; therefore, the effect of inhalational doses was not to improve *E*_max_ but the probability of target attainment (PTA) (42–44). The MIC distributions for amikacin, imipenem, and cefoxitin were those published by Ruedas-Lopez et al. (3). The tigecycline MIC distribution used was that published by Terschlüsen et al. (45).

All modeling was performed using ADAPT 5 software (46). The population PK model data for inhaled doses entered into the subroutine PRIOR for tigecycline were selected from the literature, while for imipenem and amikacin liposome inhalation suspension (ALIS), we developed the models based on publications (38–41, 46–48). ELF was specified as the direct deposition compartment of a bolus (i.e., as its own compartment). The inhalational tigecycline model was developed for our work with pulmonary *M. avium* complex disease and is described in full in that publication (49). The rest of the population PK models were generated as described next. The best models were then entered into the subroutine PRIOR of ADAPT 5.

For imipenem and ALIS, we generated the population PK model from published concentration data as a one-, two-, or three-compartment model and chose the best model using Akaike Information Criteria, Bayesian Information Criteria, and parsimony. For ALIS, Rubino et al. (38) described population PK parameters in 53 patients treated with 590 mg once each day, 14 from TR02-112 trial and 39 patients from CONVERT trial. The investigators analyzed the serum concentrations and built a three-compartment model. They also presented sputum concentrations of amikacin from the two trials, which they did not use for the population PK model. We analyzed the published sputum concentrations with the assumption that sputum concentrations mirror ELF concentrations. For imipenem, we created an inhalational PK model based on ELF and serum concentrations reported by Badia et al. (40).

In rats receiving nebulization, the cefoxitin ELF half-life was 1.54 h, while that in plasma was 1.23 h, basically similar (41). After intravenous administration, the cefoxitin ELF half-life was 0.19 h versus 0.23 h in plasma. Thus, inhaled formulations resulted in half-life at least six times longer than after intravenous, which means that the elimination rate (*k*_el_) in ELF after inhalation is six-fold lower than after intravenous administration. After nebulization, the ELF/plasma area under the curve (AUC) ratio was 1,147-fold, and the ELF/plasma peak concentrations ratio 1,000-fold (41). This means the higher AUCs and peak concentrations reflect differences in systemic versus ELF volumes. Moreover, volume scales weight-to-weight at a power of 4/4 (or 1) between rats and humans (50–52). Cefoxitin plasma/serum-based population PKs in a total of 164 critically ill patients receiving intravenous administration therapy were best described using a one-compartment model with a mean clearance and standard deviation of 10.9 ± 6.1 L/h or intra-individual (IIV) % of 55.96% and volume of distribution of 23.4 ± 10.5 L (IIV 44.87%) (47, 48). Based on these considerations, we set the cefoxitin ELF volume at 0.02L with an IIV % of 44.87% and an ELF clearance of 1.82 × 10^−3^ L/h with an IIV % of 55.96%.

For ALIS, we examined doses of 590 mg every 24 h (Q24h), Q48h, Q72h, once a week and half the dose or double the dose once a week. For imipenem, we tested doses of 1G, 2G, 3G, or 4G for both Q12h and Q24h schedules, prepared in normal saline. For cefoxitin, we examined nebulized doses of 100, 200, 400, 800, and 1,000 mg a day, prepared in normal saline. For tigecycline, the doses were 1, 2, 3, 4, 5, and 10 mg once a day, as in prior publication (49).

## RESULTS

### Literature search

**Fig 1:**
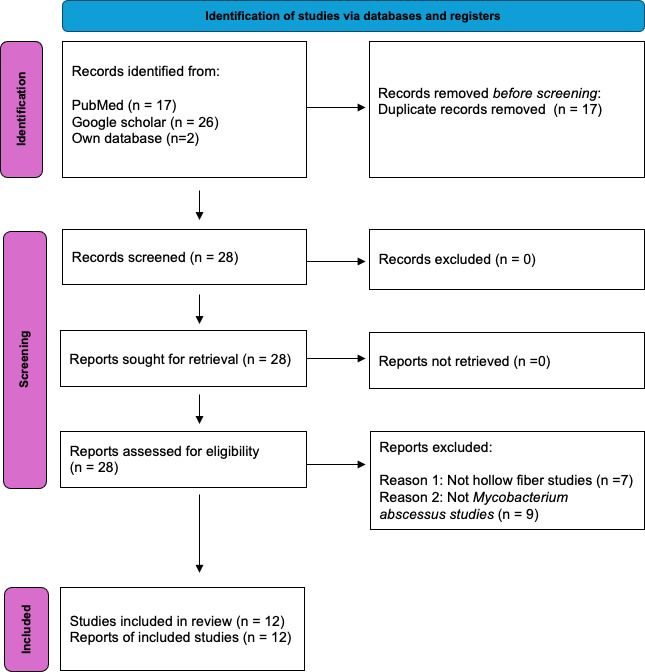
PRISMA Flow Diagram for Studies. The template used was from Page et al. (32).

The literature search is shown in Fig. 1. Out of 20 publications, 12 were classified as true HFS-MAB PK/PD studies (8, 53–63), while 8 were not (5, 29, 64–69). Ten HFS-MAC studies were monotherapy for amikacin (twice), tigecycline, moxifloxacin, omadacycline, imipenem, apramycin (compared to amikacin), epetraborole, ceftaroline-avibactam, and sulbactam-durlobactam alone and with ceftriaxone (double β-lactam). Two were combination therapy for guideline-based therapy, and two consisted of both monotherapy and combination therapy studies. Studies were performed in four different laboratories whose details are given in supplementary results. Most studies were with the ATCC reference isolate 19977 (ATCC#19977), and only recent studies included other clinical isolates.

### Quality scores

Quality scores for the studies are shown in Fig. S1A. The mean score for monotherapy studies was 13.7±1.07 out of 24, which is in the adequate category, while that for combinations was 9.75±7.41 out of 21, which is in the poor category. The most deficient scoring criteria were for the non-inclusion of non-ATCC isolates, followed by the lack of replicates. In a correlation study to identify drivers of the score (Pearson *r* >0.5 or <−0.5), shown in Figure S1B, the quality score was driven by (i) failure to utilize a *B_0_* similar to that in patient lesions, a measure of “relevance to human biology” (6), (ii) low number of exposures tested, and (iii) non-performance of MCEs. MCEs were reported in only 7 of 12 (58%) studies.

### Qualitative systemic analysis findings and real-world evidence

The studies, qualitative findings, and their quality scores are shown in Table 1. A full description of the lessons learned is in the supplementary results section. Guideline-based therapy studies demonstrated poor efficacy in HFS-MAB, as encountered in patients. Drug administration was either for 14 or 21 days. Overall, the monotherapy studies demonstrated that the HFS-MAB is tractable as a tool for exposure-effect studies, dose fractionation studies, and for comparison of combination regimens for both microbial kill and AMR. An important contribution in Table 1 was that of two studies that also presented real-world evidence clinical data versus the HFS-MAB output (56, 58). The learnings were that biologic activity in the HFS-MAB and microbial kill below *B*_*0*_ could be translated to patients’ sputum culture conversion. In addition, the sulbactam-durlobactam study was analyzed using 𝜸-slopes that were then translated to sputum culture conversion in patients, using extinction mathematics (62).

**TABLE 1:** Qualitative systematic analysis findings and quality of studies by year of publication

Drug	Year/Ref	Findings and conclusions	Real world evidence	Quality score	Study quality
Amikacin (8)	2015	First HFS-MAB published, 14-day exposure-effect study Mutation frequency to three times MIC = 2.73 ± 0.31 × 10^−6^ *C*_max_/MIC was linked to both microbial kill and AMR EC_80_ was *C*_max_/MIC = 3.2 *E*_max_ was 1.01 log_10_ CFU/mL below *B*_0_ *C*_max_/MIC linked efficacy based on inhibitory sigmoid *E*_max_ and to AMR based on antibiotic resistance of time model	None	16	Good
Tigecycline (53)	2016	21-day exposure-effect study Tigecycline unprecedented feat of >1.0 log_10_ CFU/mL below *B*_0_ Proposed optimal intravenous dose of 200 mg toxic to patients	None	13	Adequate
Moxifloxacin (54)	2016	MIC_50_ of 8 mg/L and MIC_90_ of 16 mg/L in 134 isolates First 28-day exposure-effect study and 28-day dose fractionation study Mutation frequency to three times MIC = 2.11 ± 0.16 × 10^−5^ AMR demonstrated antibiotic resistance of time model characteristics and replaced all treated systems by day 21 MCE identified dose of 800 mg; susceptibility MIC breakpoint of 0.25 mg/L versus MIC_50_ of 8 mg/L	None	11	Adequate
Guideline-based therapy (55)	2016	Exposures were clarithromycin AUC_0–24_/MIC of 5.3 (AUC_0–24_ = 42.4 mg*h/L), cefoxtin %*T*_MIC_=100, and amikacin *C*_max_/MIC = 4 (*C*_max_ = 128 mg/L) Guideline-based therapy failed after 14 days, killed 1.22 log_10_ CFU/mL below *B*_0_	Meta-analysis: 34% sputum culture conversion versus 1.22 log_10_ CFU/mL below *B*_0_	6	Poor
Omadacycline (56)	2023	MIC_50_ of 1 mg/L and MIC_90_ of 2 mg/L in 20 isolates Omadacycline killed 2.09 log_10_ CFU/mL below *B*_0_ In MCE, 300 mg oral drug achieved EC_80_ in 99.46% patients	Person, intervention, comparison, and outcome (PICO) analysis: 300 mg oral drug in combination achieved sputum culture conversion in 80% cases vs 33% comparators	10	Adequate
Amikacin (57)	2024	14-day HFS-MAB study %*T*_MIC_ linked efficacy and resistance Microbial kill calculated at 2.0 log_10_ CFU/mL below *B*_0_	None	16	Good
Imipenem (58)	2024	For MIC assays, 24-h readout in Middlebrook 7H9 broth best mitigated drug degradation in MIC assays Imipenem + relebactam MIC_50_ was 2 mg/L and MIC_90_ 4 mg/L in 122 isolates In the HFS-MAB, imipenem killed 1.32 log_10_ CFU/mL below *B*_0_ In MCE, intravenous infusion over 1 h of 500 mg imipenem + relebactam, every 6 h was optimal dose In MCE, the susceptibility breakpoint MIC was 8 mg/L	PICO analyses: days-to-sputum culture conversion were 470 in comparators, 311 for imipenem added on failing regimen, and 37 in newly treated	16	Good
Guideline-based therapy (59)	2024	HFS-MAB lasted 10 days of therapy AMR captured as growth on agar supplemented with 80 mg/L of cefoxitin (10 × MIC) and 32 mg/L of amikacin (32 × MIC) Cefoxitin PKs in lungs were a continual infusion (%*T*_MIC_ = 100), amikacin *C*_max_ = 80 mg/L, but clarithromycin AUC was not reported Amikacin, cefoxitin, and clarithromycin combination demonstrated poor activity against MAB	Yes, meta-analysis: 34% sputum culture conversion versus 0 CFU/mL kill in HFS-MAB	3	Deficient and unreliable
Apramycin (60)	2025	14-day HFS-MAB study Apramycin activity exceeded that of amikacin in two isolates *E*_max_ was 1.51 and 2.18 log_10_ CFU/mL below *B*_0_ PK/PD parameter linked to microbial kill was %*T*_MIC_	No	9	Poor
Epetraborole (EBO) (61)	2025	MIC_50_ was 0.125 mg/L and MIC_90_ 0.25 mg/L in 59 isolates 21-day exposure effect study MICs rose in all treated systems with an invented “U” curve of MIC versus AUC/MIC Resistant subpopulation linked to AUC/MIC Concentration-dependent LeuRS mutations, including novel mutations at Asp433Val, Arg324Ser, and Phe310Cys EBO killed at least 2.4 log_10_ CFU/mL below *B*_0_	No	14	Adequate
Ceftaroline-avibactam (63)	2025	MIC_50_ and MIC_90_ had a large regional variation Ceftaroline-avibactam mutation frequency was 8.83 ± 2.37 × 10^−7^ Microbial kill and AMR linked to AUC_0–24_/MIC more than to %*T*_MIC_ AMR suppression achieved at AUC_0–24_/MIC 2.569. Ceftaroline-avibactam-moxifloxacin-tigecycline demonstrated concentration-dependent efficacy, and microbial kill equaled guideline-based therapy Ceftaroline-resistant subpopulation was 41.22 times lower than cefoxitin	No	12	Adequate
Sulbactam-durlobactam ± ceftriaxone (62)	2025	Highest microbial kill seen so far in the HFS-MAB %*T*_MIC_ linked to both microbial kill and resistance Sulbactam-durlobactam plus ceftriaxone killed without regrowth and demonstrated Bliss’ additivity. ***γ*** of bacterial population in virtual human subjects was 2.28 (0.97–4.80) log_10_ CFU/mL/day for sulbactam-durlobactam-ceftriaxone and 2.91 (1.65–4.93) log_10_ CFU/mL/day for sulbactam-durlobactam-ceftriaxone plus epetraborole plus omadacycline.	No	20	High

### Quantitative analysis and ranking of drugs by kill below *B_0_*

In Table 2 and Fig. S2, all drugs were ranked by efficacy. Sulbactam-durlobactam-ceftriaxone was ranked highest, epetraborole was ranked second, and omadacycline was ranked third. The PK/PD target exposures are also shown in Table 2.

**TABLE 2:** Quantitative systemic analysis findings and ranking of efficacy of drugs for monotherapy

Drug (reference)	*E*_max_ (log_10_ CFU/mL) kill below *B*_0_	Fold of 3-drug guideline-based therapy kill below *B*_0_	Rank	PK/PD target	Dose schedule (oral and IV)	PK/PD MIC breakpoint (mg/L)
Monotherapy						
Sulbactam-durlobactam/cefriaxone (62)	3.47	176.8	1	%*T*_MIC_ = 50%	2G q8h for CrCl^*a*^ of >90 mL/min. 2G q12h for CrCl of 60–90 mL/min. 1G q12h for CrCl of ≥30 to <60 mL/min. 1G q24h for CrCl of <30 mL/min.	
Epetraborole (61)	2.44	14.76	2	AUC_0–24_/MIC = 202.73	Once daily or twice daily	0.5
Omadacycline (56)	2.08	7.24	3	AUC_0–24_/MIC = 23.76	Once daily	1.0
Apramycin (60)	2.07	6.85	4	Too few exposures to calculate PK/PD target	Not clear	None
Amikacin (average of 4 weighted by score) (8, 57, 60)	1.6	2.42	5	*C*_max_/MIC = 3.2; %*T*_MIC_ = 40%	Liposomal amikacin, once daily to once weekly	8
Tigecycline (53)	1.3	1.47	6	AUC_0–24_/MIC = 36.65	Once daily	0.5
Imipenem/relebactam (58)	1.23	1.00	7	%*T*_MIC_ = 48%	1 h infusion every 6 h	8
Ceftaroline/avibactam (63)	0.69	0.29	8	AUC_0–24_/MIC = 9.84	Twice daily	
Moxifloxacin (54)	0	0	9	AUC_0–24_/MIC = 102.11	Once daily	0.25
Combination therapy						
Guideline-based therapy 1 (55)	1.23	1	Ref	Not applicable	Different for each drug	Not applicable
Guideline-based therapy 2 (59)	0	0		Not applicable	Different for each drug	Not applicable
Guideline-based therapy 3 (63)	1.20	0.94		Not applicable	Different for each drug	Not applicable
Ceftaroline/avibactam-moxifloxacin-tigecycline (63)	0.48	0.18		Not applicable	Different for each drug	Not applicable

^*a*^ CrCl, creatinine clearance.

### MCEs with new inhalational formulations

**Fig 2:**
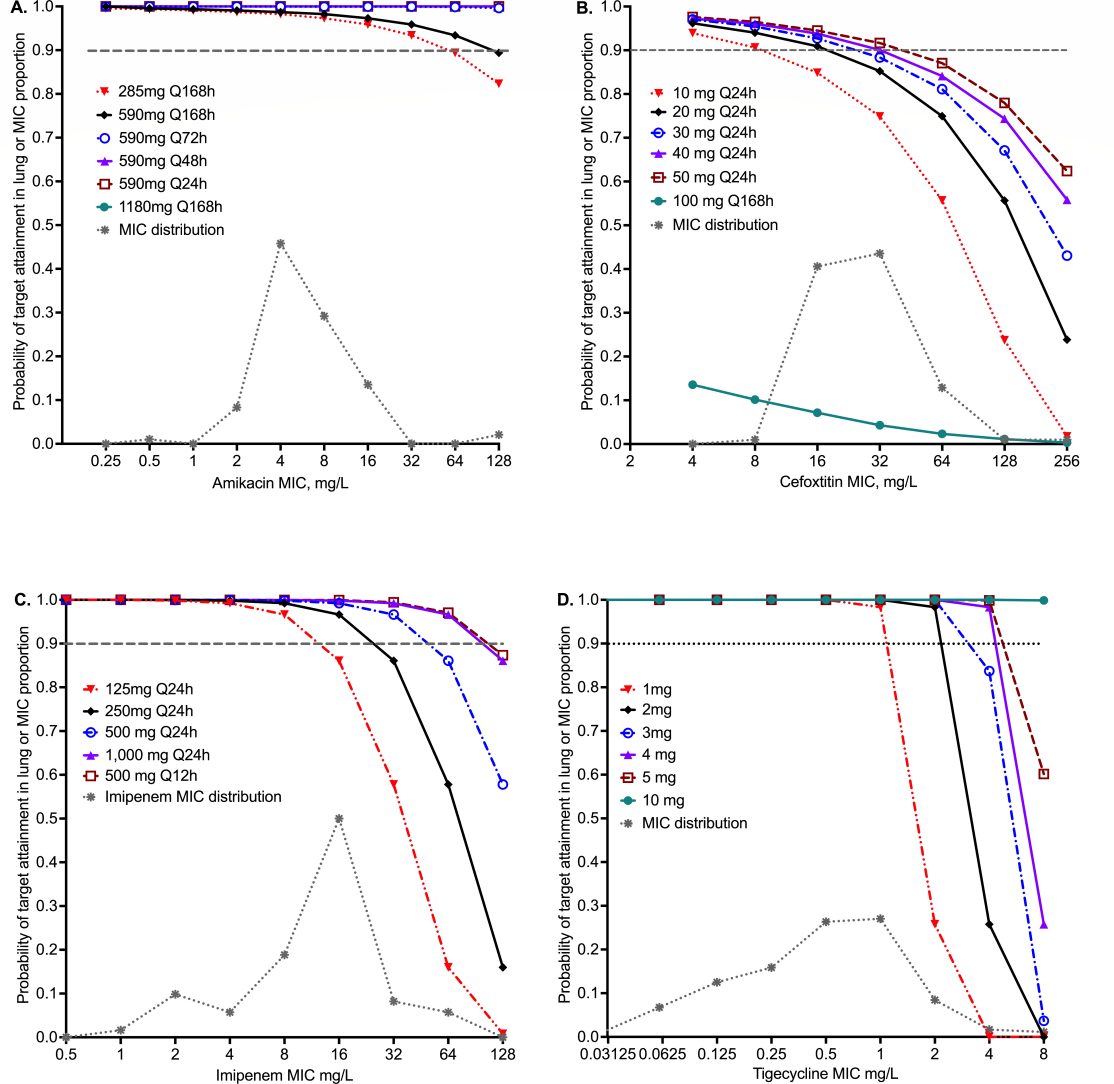
PTA for inhaled amikacin, cefoxitin, imipenem, and tigecycline doses and schedules. PTA for each dose and dosing schedule is shown at each MIC. The symbols indicate PTA estimates for 10,000 patients. The high concentrations achieved by all the drugs in the lungs reflect the low ELF volume, compared to the systemic circulation. (**A**) Amikacin PTA shows that 590 mg of ALIS daily, every other day, and every 72 h achieved the PTA of ~100% across the range of MICs measured. The once-a-week regimen falls below 90% at an MIC of 256 mg/L. (**B**) Cefoxitin, which has a half-life of about 1 h in serum, achieves high concentrations in ELF and is cleared six times slower (41). We assumed a target of 100% *T*_MIC_. (**C**) The imipenem MIC_90_ was 32 mg/L, and once a day, doses ≥500 mg achieved PTA of >90% at this MIC. (**D**) Low doses of inhaled tigecycline such as 2 mg achieved PTA of >90% above the MIC_90_.

For the ALIS population PK parameters, we chose a three-compartment model as shown in Table S1. The amikacin population PK parameter estimates of the three-compartment model entered into the domain of input are shown in Table 3. Figure S3 shows the amikacin concentration-time profiles for the five ALIS doses and dosing schedules during the first 7 days. During the first 72 h, the predicted median sputum concentrations versus those observed by Rubino et al. (38) overlapped, validating our model. The PTAs for all ALIS doses and dosing schedules tested using the *C*_max_/MIC target of 3.2 of Ferro et al. (8) was 100% up to an MIC of 128 mg/L. Figure 2A shows that all doses and schedules achieved the 40% *T*_MIC_ target exposure from Gibson et al. (57) in more than 90% of patients until an MIC of 128 mg/L, except 295 mg once a week. Thus, the amikacin PK/PD MIC breakpoint for inhaled ALIS was determined as 256 mg/L, which is higher than for intravenous dosing in Table 2.

**TABLE 3:** Population PK parameter estimates

	Subroutine PRIOR		10,000 virtual subjects	
	Estimate	% CV	Estimate	% CV
ALIS (38)				
Clearance in ELF compartment in L/h	0.16 × 10^−2^	71.8	0.16 × 10^−2^	70.82
Volume in ELF compartment in L	0.08	35.1	0.08	34.88
Clearance in central compartment in L/h	0.02	71.8	0.01	72.29
Volume in central compartment in L	0.05	65.09	0.05	65.1
Clearance in peripheral compartment in L/h	1.99	30.24	1.05	29.78%
Volume in peripheral compartment in L	3.25 × 10^−4^	40	3.26 × 10^−4^	39.56
Cefoxitin (41, 47, 48)				
Clearance in ELF compartment in L/h	1.82 × 10^−3^	55.96	1.82 × 10^−3^	55.96
Volume in ELF compartment in L	0.02	44.87	0.02	44.41
Imipenem (40, 46)				
Clearance in ELF compartment in L/h	1.54 × 10^−1^	100	1.54 × 10^−1^	98.15
Volume in ELF compartment in L	0.06	69.4	0.06	70.87
Intercompartmental clearance L/h	7.80	110	7.82	107.6
Peripheral volume (L)	11.1	56	11.03	56.49

Figure S4 shows the steady-state cefoxitin concentration-time profiles for six inhalation doses. According to the FDA package insert, 1,000 mg of intravenous cefoxitin achieves a *C*_max_ of 110 mg/L, which means *C*_max_ with 10 mg would be around 1.1 mg/L versus 516.6 mg/L with the same dose via inhalation route (70). Figure 2B shows the PTAs for the cefoxitin doses and schedules. The PK/PD target for cefoxitin has not been identified, and we assumed a worst-case scenario of 100% *T*_MIC_. The PTA for a 50 mg dose was 87% at the MIC_90_ of 64 mg/L; hence, this is the cefoxitin susceptibility MIC breakpoint with nebulization.

Regarding imipenem, the best scores were for a two-compartment PK model, as shown in Table S2. The population PK parameter estimates in the domain of input derived from the literature were compared to those in 10,000 virtual subjects in Table 3; Fig. S5 shows the steady-state imipenem concentration-time profiles for the five inhalation doses and schedules. Figure 2C shows the PTAs for six doses and schedules, for %*T*_MIC_ of 48% (58). The PTA for 250 mg once a day dose fell to 86% at the MIC_90_ of 32 mg/L and is the imipenem susceptibility MIC breakpoint with inhalation.

Tigecycline concentration-time curves were shown in the original paper where the model was developed for *M. avium* LD (49). Figure 2D shows the PTAs and demonstrates that even at as low doses as 2 mg, >90% of virtual patients with isolates at the MIC_90_ of 2 mg/L achieved the PK/PD target. At the dose of 4 mg, the tigecycline susceptibility MIC breakpoint after inhalation was 8 mg/L, much higher than the 0.5 mg/L after intravenous (IV) dosing in Table 2.

**Fig 3:**
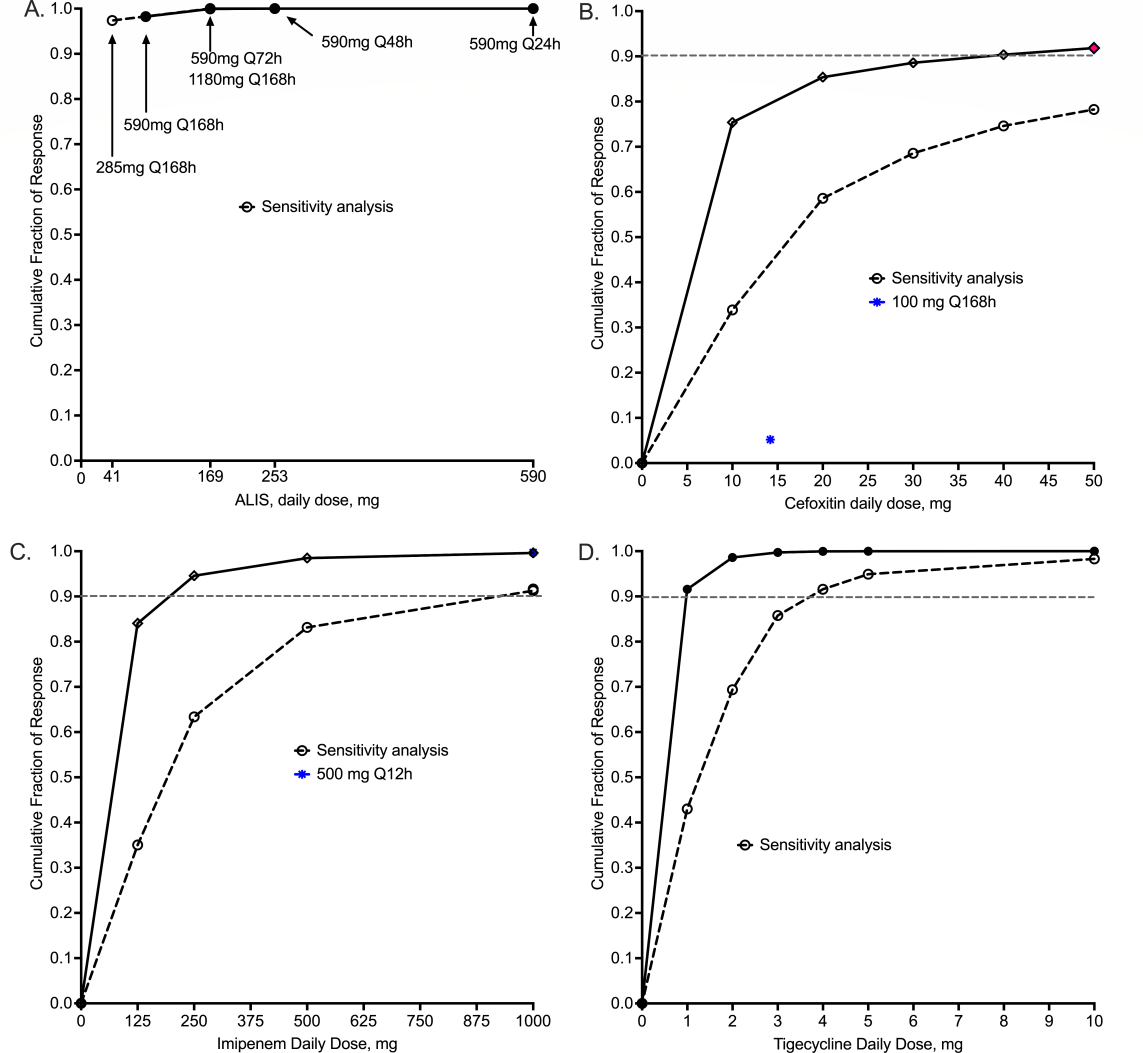
Cumulative fraction of response for 10,000 patients at each dose. Sensitivity analysis was made by shifting MICs by two tube dilutions up (that is making the isolates more resistant than observed). Symbols are point estimates for 10,000 virtual subjects. (**A**) All doses of ALIS tested achieved a cumulative fraction of response (CFR) of >90%, including the once-a-week dosing. Sensitivity analysis did not change the final estimates. (**B**) The cefoxitin dose of 50 mg achieved CFR in >90% of patients; sensitivity analysis revealed a CFR of 80%. (**C**) The imipenem daily dose 250 mg achieved a CFR of >90% but falls to 60% on sensitivity analysis. However, given that, unlike our sensitivity analysis, the MICs are likely lower than observed because of imipenem degradation in the MIC assays (58), the dose of 250 mg is recommended. (**D**) Sensitivity analysis leads to a tigecycline dose of 4 mg being sufficient minimal inhaled dose for treatment of pulmonary MAB.

The cumulative fraction of response for the four drugs is shown in Fig. 3. The following inhalational doses were identified as optimal for MAB-LD, 590 mg once weekly for ALIS (Fig. 3A), 50 mg/day for cefoxitin (Fig. 3B), 250 mg/day for imipenem (Fig. 3C), and 4 mg/day for tigecycline (Fig. 3D).

## DISCUSSION

The HFS-MAB fits the FDA roadmap and EMA process to replacing animal testing in preclinical studies with scientifically validated NAMs (6, 7). However, the quality scores were in the range that can be improved upon. Drivers of deficient scores inform us of what needs to be improved and to be standardized. First was the inclusion of four to five isolates representative of the majority of clinical isolates from patients, to allow for more robust PK/PD target setting (33). This is also important for the generalization of PK/PD study findings. Second, was lack of replicates. Replicates provide important quality controls on the conduct of experiments. Therefore, we recommend at least two replicates in exposure-effect and dose-fractionation studies and at least three in combination therapy studies. Third, there was failure to utilize a *B*_*0*_ similar to patient lesions, which is one of the most important predictors of efficacy (10, 13–17). The remedy is the use of a higher inoculum so that total *B*_*0*_ per HFS-MAB cartridge is 10^7^–10^8^ CFU (10, 13). Fourth was the number of exposures tested in exposure-response studies. The best number of doses is seven to eight, including at least one on the steep portion of the dose-response curve. Finally, this also calls for standardization of this model between laboratories, including related assays such as MICs, inoculum preparation, and best sampling days.

HFS-MAB work in four laboratories shows that this tool can be used by multiple groups. Even as the model continues to be improved, here, we showed that the HFS-MAB is tractable, with successful completion of multiple exposure-effect and dose-fractionation studies. This model is important for setting PK/PD targets of the new/repurposed drugs, from which clinical doses and susceptibility breakpoints need to be set. The HFS-MAB was also used for combination therapy studies. This is best done via the factorial design of EC_20_, EC_50_, and EC_80_ exposures. We also learned that the biological signal of kill below *B*_*0*_ in the HFS-MAB informed on sputum culture conversion in patients, based on two real-world evidence studies (56, 58). While more work will be required, this type of translation from HFS-MAB to patients is promising (56, 58). On the other hand, given the variation of kill rates between laboratories, we recommend standardized guideline-based therapy (intrapulmonary PKs of intravenous amikacin plus cefoxitin plus oral clarithromycin) as a positive control in all HFS-MAB studies. If the guideline-based therapy and non-treated HFS-MAB behave differently from historical controls, then the study quality would be considered compromised. This should improve reproducibility.

AMR abrogates the effect of guideline-based therapy in patients (71). In the HFS-MAB studies in Table 1, AMR also universally terminated microbial kill. MAB has a diverse armamentarium of AMR mechanisms targeting both old/repurposed and new drugs (29, 61, 72). Because repetitive sampling is the *modus operandi* in the HFS-MAB, this enables tracing the evolution of AMR under different drug exposures, over time, as is the case with patients’ sputa. Importantly, the HFS-MAB is a preclinical drug development tool in which combination therapy can be tested for the ability to abrogate AMR, to develop regimens that will not fail when used in patients.

MAB-LD is an orphan disease; therefore, the number of participants available for MAB clinical trials will be limited. In this context, the HFS-MAB is an agnostic platform to rank monotherapy drugs and combinations, at optimal exposure for each drug, by how well they will perform relative to guideline-based therapy. This allows choice of drugs for combination therapy based on extent of kill below *B*_*0*_. Based on this approach, the three top-ranked were sulbactam-durlobactam/ceftriaxone, epetraborole, and omadacycline. We recommend these drugs be combined in factorial design (using EC_20_, EC_50_, and EC_80_) in the HFS-MAB and compared to guideline-based therapy. The result could be a novel regimen to test versus guideline-based therapy in a 6-month open-labeled clinical trial with 𝜸-slopes and time-to-sputum culture conversion outcomes (62, 73–76). This avoids the clinical trial design paradigm of iterative switching one new drug into guideline-based therapy at a time in randomized trials versus guideline-based therapy, stretching out the time to a novel regimen into a decade.

Antimicrobial PK/PD studies are important because they identify the exposure target to be achieved by differing doses in MCEs (77). Why would we do exposure-response PK/PD studies if the aim is not to find a target exposure for use in dose finding? Thus, we recommend MCEs to be performed in tandem with every HFS-MAB study. As an example, here, we examined the inhalation doses of four drugs that are currently administered intravenously, some administered multiple times a day. The ALIS results demonstrated that 590 mg a day, the dose used in clinical trials such as the CONVERT study (78), would achieve the target exposures in >99% of patients. Indeed, a once-a-week dosing schedule for the same dose was shown to be optimal as well, which may improve patient compliance. We also report new doses for cefoxitin, imipenem, and tigecycline inhalational formulations. Thus, the HFS-MAB was useful in identifying dosing for new drugs and new formulations. Part of the learnings were to identify greater than five doses *in silico*, as well as multiple dosing schedules, via different routes of administration for efficacy versus toxicity in the MCEs.

There are several caveats to our MCEs. First are the population PK parameter estimates in ELF assumptions. For some drugs, these parameters may not be accurate, especially when the underlying PK model is derived from small patient populations or sparse sampling. Parameter estimates such as ELF volumes and systemic absorption from the lungs can be problematic if not obtained from intensive sampling using bronchoalveolar lavage in patients. Second, amikacin sputum concentrations were assumed to be reflective of ELF concentrations but are likely an underestimate given the possible dilution of sputum with saliva. Third, for cefoxitin, we extrapolated from studies in rats, especially as regards to drug serum versus ELF half-life, and assumed the same ratios in patients. The tigecycline PK parameter estimates were also based on physiologically based PK modeling from animal to human lung models. These assumptions are critical to the veracity of the MCE-derived doses and point to important caveats for clinicians who may be considering using these doses.

### Limitations in performance of systematic analyses

First, inherent in all meta-analysis is that systematic errors in original studies are carried forward in pooled meta-analyzes studies. This is an important confounding factor. Second, the search strategy resulted in inconsistent findings between the investigators, solved by conference. This is likely because MeSH headings and keywords in the HFS-MAB field are not standardized, so that we could have missed some publications. Third, different outcome definitions (e.g., raw CFU/mL versus kill below *B*_*0*_), sampling schedules, laboratory assays, and laboratory protocols were used by the different laboratories and are a confounding factor in performance of the quantitative meta-analysis. These confounding factors will be addressed in work that focuses on standardizing the HFS-MAB protocols.

## Data Availability

The data for the results presented in the manuscript are publicly available.

## SUPPLEMENTAL MATERIAL

Supplemental material spectrum.03296-25-s0001.pdf Supplemental methods; Fig. S1 to S5; Tables S1 and S2.

ASM does not own the copyrights to Supplemental Material that may be linked to, or accessed through, an article. The authors have granted ASM a non-exclusive, world-wide license to publish the Supplemental Material files. Please contact the corresponding author directly for reuse.
