# Supplementary material for: Systematic and quantitative analyses of hollow fiber model of Mycobacterium abscessus lung disease studies and new dosing recommendations
Source: Microbiol Spectr. 2026 Apr 21;14(6):e03296-25. doi: 10.1128/spectrum.03296-25 (PMC13228079; doi:10.1128/spectrum.03296-25)
Supplement: Supplemental material — Supplemental methods; Fig. S1 to S5; Tables S1 and S2. [file spectrum.03296-25-s0001.pdf]

Systematic and quantitative analyses of hollow fiber model of *Mycobacterium abscessus* lung disease studies.

Shashikant Srivastava and Tawanda Gumbo

SUPPLEMENTRAY DATA

Supplementary Methods

Supplementary References

Supplementary Results Figures

Supplementary Results Tables

SUPPLEMENTARY METHODS

STUDY QUALITY SCORING

Criterion	No	1	2	3	4	5	Total Possible Score
**EXPOSURE EFFECT**							
B_0 :<br>0 score if not reported<br>1 if density is reported but is outside the range seen in patients<br>2 if reported and within range encountered in patients<br>3 if both density and total bacterial burden are reported	0	1	2	3			3
Number of exposures:<br><5 exposures =0,<br>5 exposures =1<br>6 exposures =2<br>7 exposures =3<br>8 exposures =4<br>At least one exposure of steep portion = 1 extra point	0	1	2	3	4	5	5
Dose fractionation study was performed: 0 if non, 2 if fractionation for at least 2 dose, 3 if at least 3 doses fractionated	0		2	3			3
MCE: score by number of doses tested	0	1	2	3	4	5	5
Number of non-lab-reference (ATCC) isolates for PK/PD target	0	1	2	3	4	5	5
Number of replicates:<br>One =0<br>2 replicates=1<br>3 replicates in dose response=2<br>If more than 1 replicate for both reference lab strain and the clinical isolates = 3	0	1	2	3			3
**Total Possible Score**							**24**
**COMBINATION** (similar to exposure-effect but replace [1] number of exposures, [2] dose-fractionation, and [3] MCE with-							
Score:<br>0 if one dose/exposure of each drug was used,<br>3 if at least 3 non-zero exposures were used for each drug,<br>4 if 2 replicates were used, and 5 if at least 3 replicates were used.	0	1	2	3	4	5	5
Score:<br>0 if there is no synergy or antagonism interaction index<br>3 if there is an interaction index calculated,<br>4 if the optimal exposures of each component for microbial kill calculated,<br>5 if optimal exposures are calculated for both microbial kill and resistance suppression	0	1	2	3	4	5	5
**Total Possible Score**							**21**

SUPPLEMENTARY RESULTS

Laboratories where HFS-MAB work was performed

The first Laboratory was that led by TG which started at the University of Texas Southwestern Medical Center, then moved to Baylor University Medical Center, and then Praedicare Inc, all in Dallas, Texas, USA. The second laboratory was that led by SS, with is at University of Texas at Tyler, Tyler, Texas, USA. The third laboratory was that led by Joy E. Gibson at the Saban Research Institute, Children's Hospital Los Angeles and the University of Southern California Keck School of Medicine, both in Los Angeles, California, USA. The fourth laboratory was that led by Elisabeth Hodille, at Laboratoire de Biologie Médicale Multi-Site, Hôpital de la Croix Rousse, Hospices Civils de Lyon and CIRI-Centre International de Recherche en Infectiologie, Ecole Normale Supérieure de Lyon, Université Claude Bernard Lyon, Inserm U1111, both in Lyon, France.

Qualitative Systemic analysis findings and real-world evidence

The studies, qualitative findings, and their quality scores are shown in **Table 1**. Drug administration was either for 14 days or 21 days. The first HFS-MAB was for amikacin and was published in 2015 (1). Since then, there have been two other amikacin studies which demonstrated a 10-fold difference in the extent of microbial kill (1-3). One study found C_{max}/MIC linked effect with an EC_{80} of 3.2, while the other demonstrated $\%T_{MIC}$ linked effect with an EC_{80} of 40% (1, 2). There were three guideline-based therapy studies which we compared in **Table 1**; two demonstrated virtually the same microbial kill below B_0, while the third study demonstrated no microbial kill below B_0 (4-6). Nevertheless, all three guideline-based therapy studies demonstrated poor efficacy in HFS-MAB, as compared to 34% sputum culture conversion in

patients. AMR developed in all twelve studies. One study used the term “tolerance” for the AMR; however, the authors did not investigate if this was true tolerance or constitutive induction of efflux pumps which is part of the antibiotic resistance arrow of time (7). Overall, the monotherapy studies demonstrated that the HFS-MAB is tractable as a tool for [1] exposure-effect studies, [2] dose fractionation studies, and [3] comparison of combination regimens, with the PD output of both microbial kill and AMR.

An important contribution in **Table 1** was that of two studies that also presented real-world evidence clinical data versus the HFS-MAB output (8, 9). Guidelines use the PICO (Population, Intervention, Comparators, Outcomes) approach for real-world evidence, an approach that was used in tandem with the two HFS-MAB studies (8-10). In the first, omadacycline killed 2.08 $\log_{10}$ CFU/mL below B_0 in the HFS-MAB (that is 7.2-fold better than guideline-based therapy in the HFS-MAB) (8). The HFS-MAB-derived EC_{80} was used in MCEs and the omadacycline standard 300 mg/day oral dose was identified as optimal. In the PICO analyses omadacycline-based combination therapy achieved sputum culture conversion that was 8-fold better than in comparator patients on guideline-based therapy that also included tigecycline. In addition, the faster time to sputum culture conversion was confirmed in prospective trialing in one patient for salvage therapy (8). Similarly, imipenem killed 1.23 $\log_{10}$ CFU/mL below B_0 in the HFS-MAB, Monte Carlo experiments identified an optimal dose and dosing schedule, and real-world evidence revealed that imipenem-based combination regimens achieved a sputum culture conversion of 83% in treatment naïve patients, 43% in treatment experienced patients, versus 29% in comparators on guideline-based therapy (9). The learnings were that biologic activity in the HFS-MAB, and microbial kill below B_0 could be translated to patients, based on real-world evidence.

Figure S1. Quality Score of HFS-MAB Studies in the Literature.

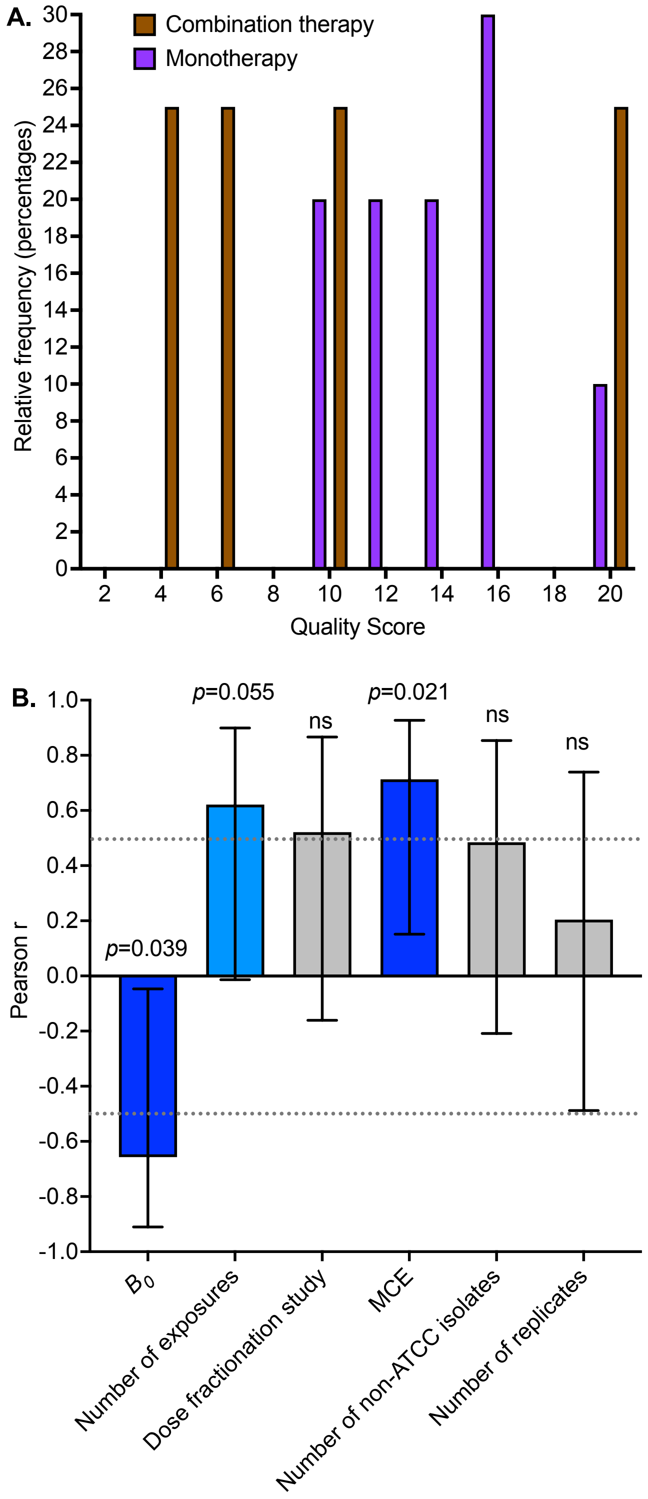

A. Distribution of quality scores shows that the majority of studies had a score below the adequate category (score<15), with at least one study in the deficient category (score <5). **B.**

Error bars are 95% confidence intervals. Hatched line is the Spearman r of 0.5 or -0.5, which we used as threshold choosing drivers of quality score. Statistically significant drivers of the scores are shown in shades of blue.

Supplementary Figure S2. Ranking of drugs by microbial kill below B_0.

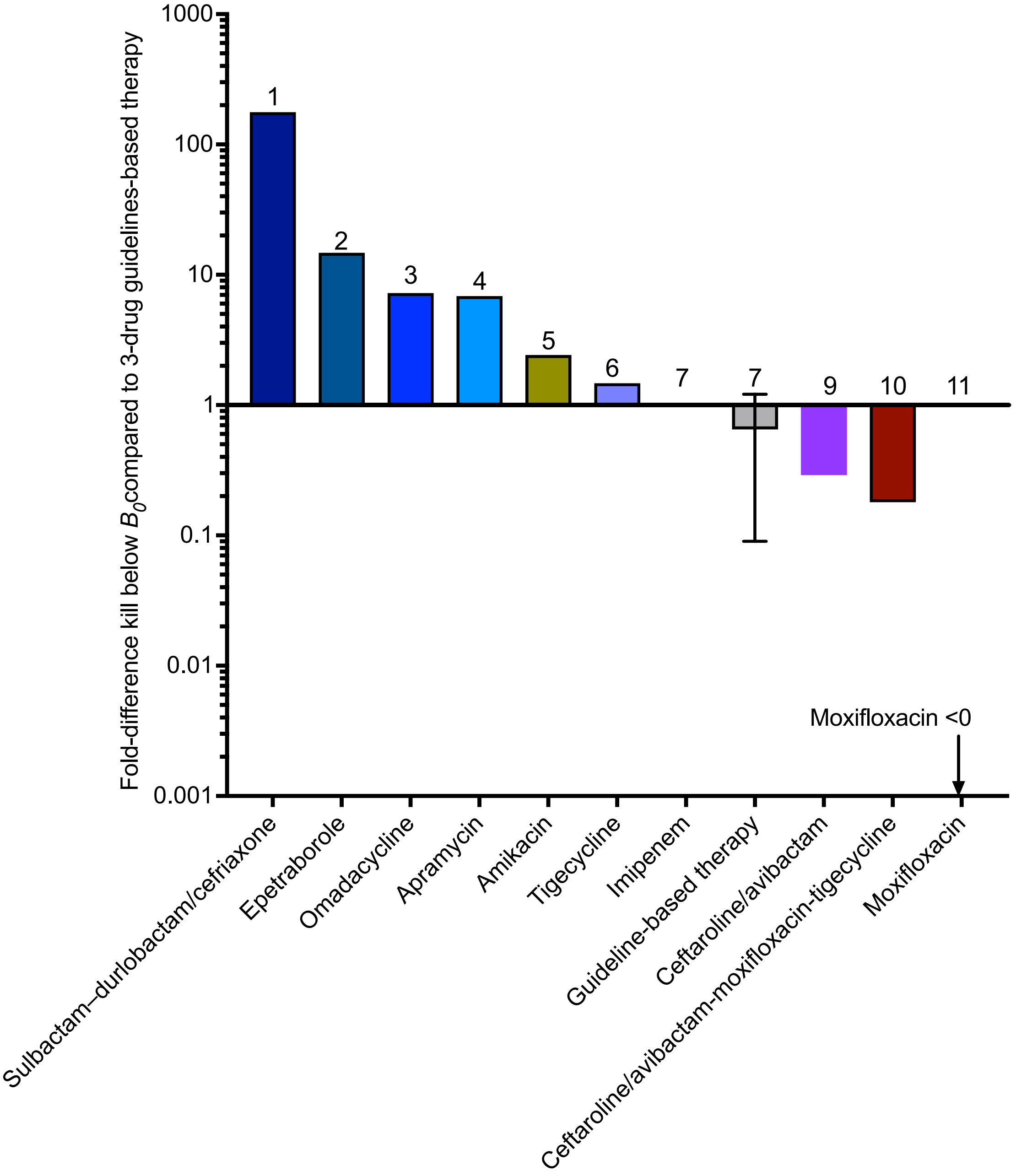

Error bar is standard deviation of the three guidelines-based therapy studies. Numbers above the bar indicate the the ranks.

Figure S3. Amikacin Liposome Inhalation Suspension (ALIS) concentration time profiles in lung

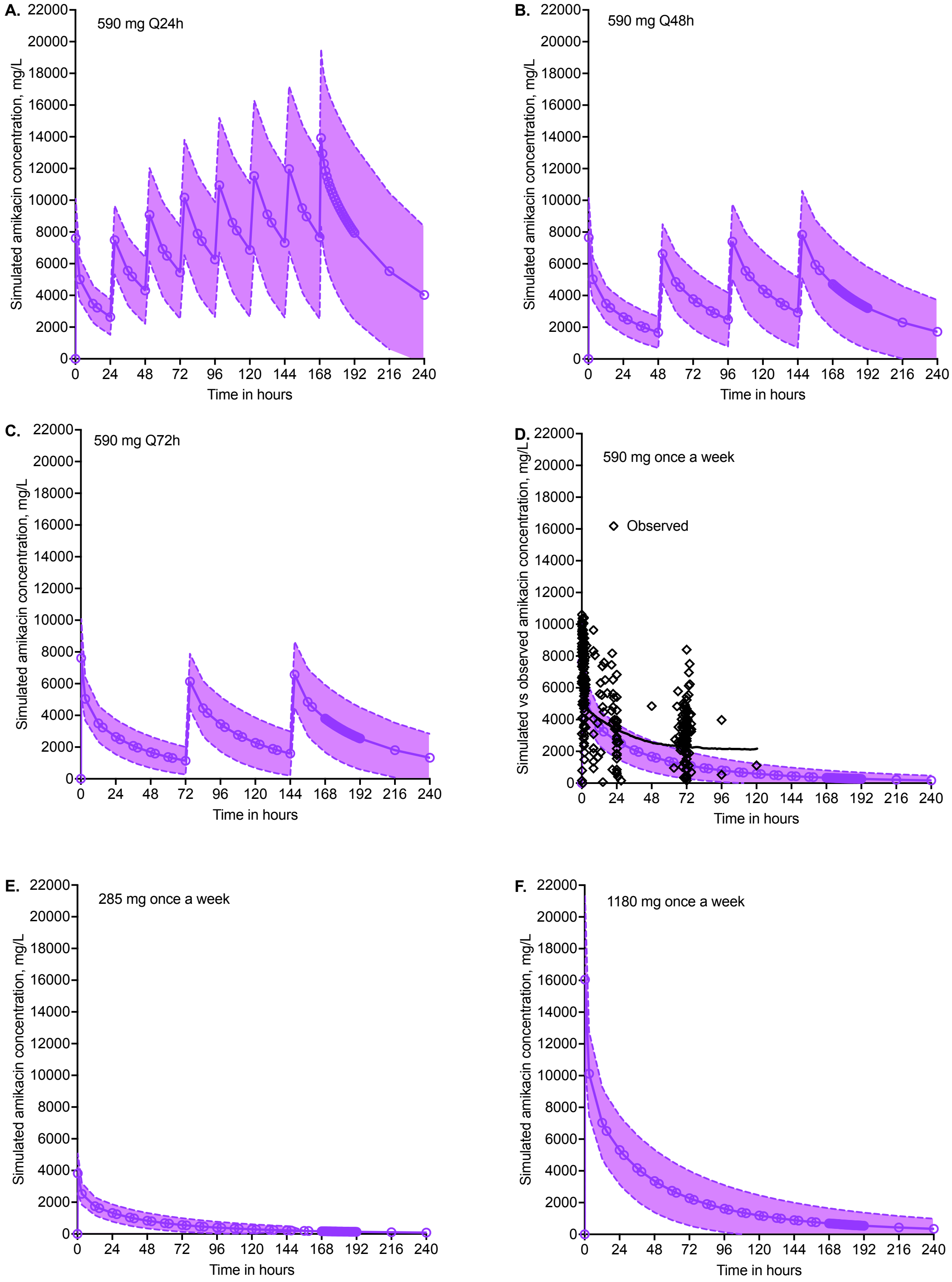

Symbols are mean concentrations in epithelial lining fluid (ELF), while shaded area is standard deviation. **A.** Standard dose administered once each day. **B.** Standard dose administered every other day. **C.** Standard dose administered every 3 days. **D.** Standard dose administered once a week versus concentrations observed in sputum. Our model had slightly faster clearance but overlapped within the first 72h. **E.** Half the standard dose administered once a week. **E.** Two times the standard dose administered once a week.

Figure S4. Cefoxitin concentration time profiles in lung

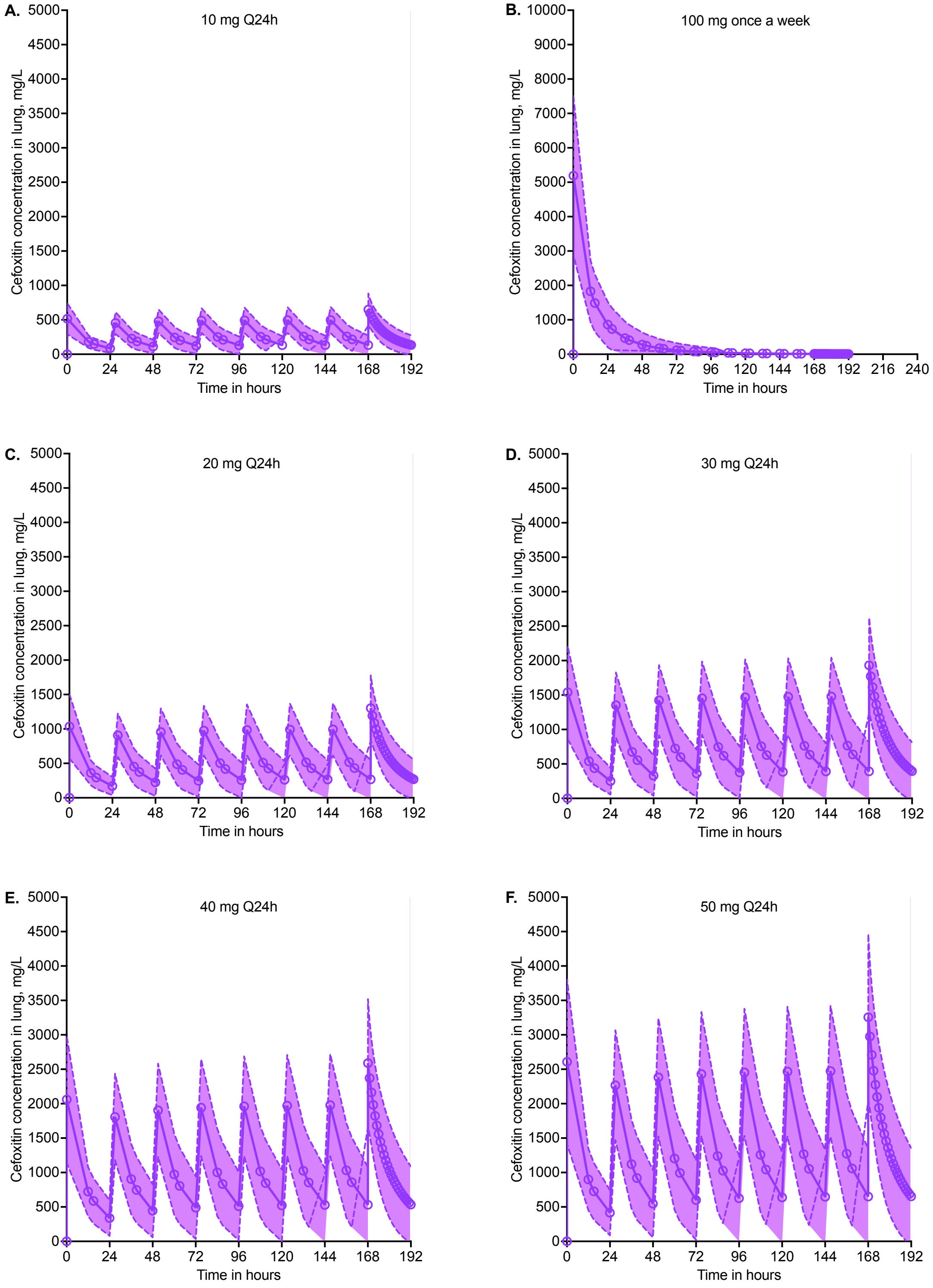

Symbols are mean concentrations in epithelial lining fluid (ELF), while shaded area is standard deviation. All doses were administered once a day.

Figure S5. Imipenem concentration time profiles in lung

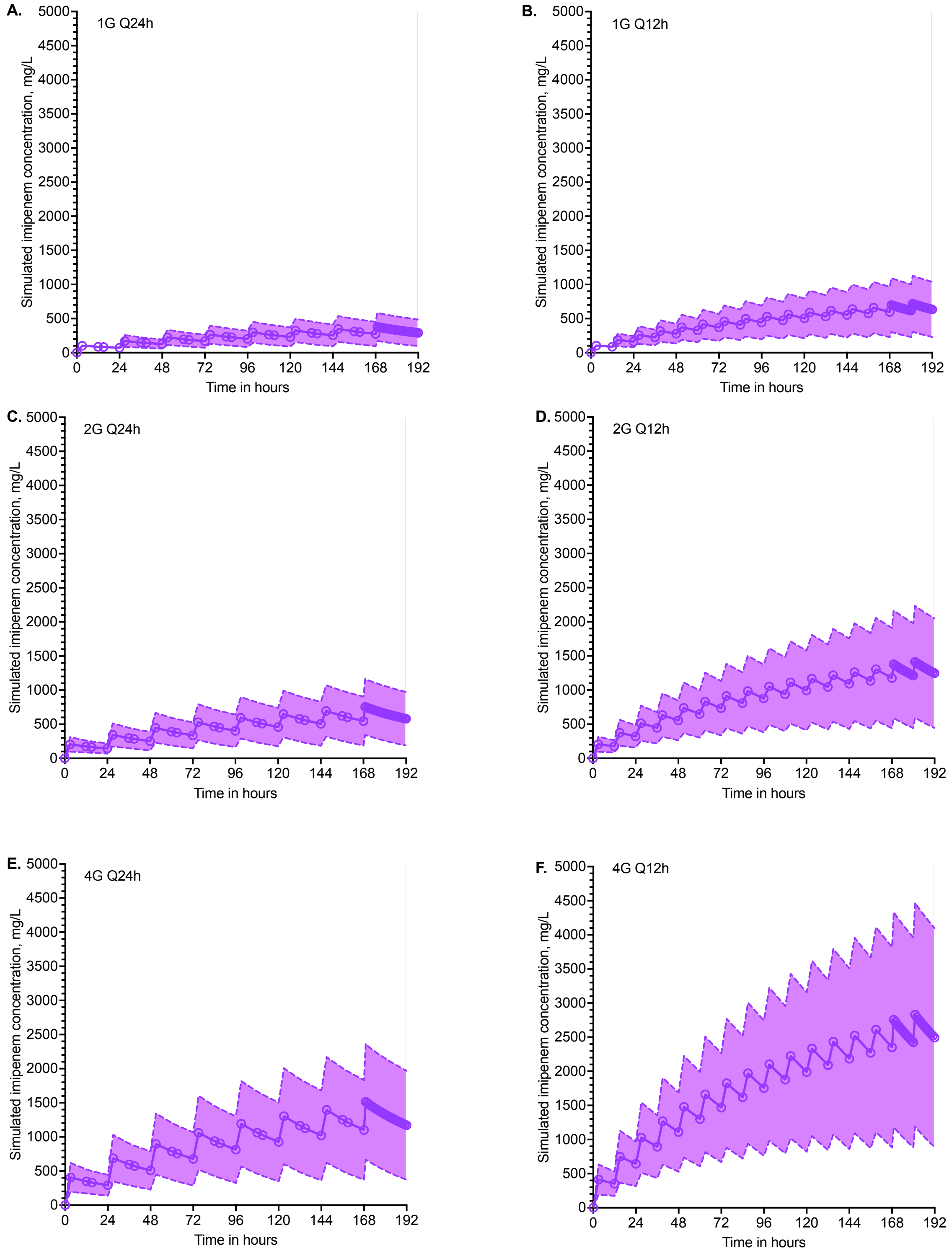

Symbols are mean concentrations in epithelial lining fluid (ELF), while shaded area is standard deviation. **A.** Imipenem 1G once each day. **B.** Imipenem 1G twice a day. **C.** Imipenem 2G once

each day. **D.** Imipenem 2G twice a day. **E.** Imipenem 4G once each day. **E.** Imipenem 4G twice a day.

Supplementary Table S1. Amikacin Population Pharmacokinetic Model Comparisons

Compartments	Akaike Information Criteria	Bayesian Information Criteria	r^2
One	1812.16	1822.58	0.770
Two	1818.16	1836.39	0.770
Three	1741.47	1764.91	0.958

Supplementary Table S2. Imipenem Population Pharmacokinetic Model Comparisons

Compartments	Akaike Information Criteria	Bayesian Information Criteria	r^2
One	83.67	85.24	0.146
Two	22.24	20.67	0.99
Three	26.24	23.89	0.99

REFERENCE

1. Ferro BE, Srivastava S, Deshpande D, Sherman CM, Pasipanodya JG, van Soolingen D, Mouton JW, van Ingen J, Gumbo T. 2015. Amikacin pharmacokinetics/pharmacodynamics in a novel hollow-fiber *Mycobacterium abscessus* disease model. Antimicrob Agents Chemother 60:1242-8.

2. Gibson JE, Nandanwar N, Neely MN. 2024. Time-dependent pharmacodynamics of amikacin on Mycobacterium abscessus growth and resistance emergence. Microbiol Spectr 12:e0322223.

3. Singh N, Dangi B, Johnson JJ, Louie A, Karunanidhi A, Curry BN, Mitarai S, Daley CL, Hobbie SN, Bulman ZP. 2025. Pharmacodynamic assessment of apramycin against Mycobacterium abscessus in a hollow fibre infection model. Journal of Antimicrobial Chemotherapy 80:1309-1314.

4. Ferro BE, Srivastava S, Deshpande D, Pasipanodya JG, van Soolingen D, Mouton JW, van Ingen J, Gumbo T. 2016. Failure of the amikacin, cefoxitin, and clarithromycin combination regimen for treating pulmonary *Mycobacterium abscessus* infection. Antimicrob Agents Chemother 60:6374-6.

5. Vignaud E, Goutelle S, Genestet C, Guitton J, Cohen S, Bourg C, Durand A, Lebouteiller L, Bernard A, Richet C, Dumitrescu O, Hodille E. 2025. Poor efficacy of the combination of clarithromycin, amikacin, and cefoxitin against Mycobacterium abscessus in the hollow fiber infection model. Ann Clin Microbiol Antimicrob 24:10.

6. Ferro BE, Srivastava S, Gumbo T. 2025. Ceftaroline-avibactam pharmacokinetics/pharmacodynamics in the hollow fiber model of Mycobacterium abscessus lung disease. International Journal of Tuberculosis and Lung Disease.

7. Schmalstieg AM, Srivastava S, Belkaya S, Deshpande D, Meek C, Leff R, van Oers NS, Gumbo T. 2012. The antibiotic resistance arrow of time: efflux pump induction is a general first step in the evolution of mycobacterial drug resistance. Antimicrob Agents Chemother 56:10.

8. Singh S, Wang JY, Heysell SK, McShane PJ, Wadle C, Shankar P, Huang HL, Pasipanodya J, Boorgula GD, Philley JV, Gumbo T, Srivastava S. 2023. Omadacycline pharmacokinetics/pharmacodynamics in the hollow fiber model and clinical validation of efficacy to treat pulmonary Mycobacterium abscessus Disease. Int J Antimicrob Agents doi:10.1016/j.ijantimicag.2023.106847:106847.

9. Singh S, Gumbo T, Wang JY, Boorgula GD, Burke A, Huang HL, McShane PJ, Amaro-Galvez R, Gross JE, Aryal S, Heysell SK, Srivastava S. 2024. Imipenem pharmacokinetics/pharmacodynamics in preclinical hollow fiber model, dose-finding in virtual patients, and clinical evidence of efficacy for Mycobacterium abscessus lung disease. J Infect Dis doi:10.1093/infdis/jiae601.

10. Daley CL, Iaccarino JM, Lange C, Cambau E, Wallace RJ, Andrejak C, Bottger EC, Brozek J, Griffith DE, Guglielmetti L, Huitt GA, Knight SL, Leitman P, Marras TK, Olivier KN, Santin M, Stout JE, Tortoli E, van Ingen J, Wagner D, Winthrop KL. 2020. Treatment of Nontuberculous Mycobacterial Pulmonary Disease: An Official

ATS/ERS/ESCMID/IDSA Clinical Practice Guideline: Executive Summary. Clin Infect Dis doi:10.1093/cid/ciaa241.
